# Eye-Strain in Health and Disease

**Published:** 1897-09

**Authors:** 


### Book Reviews.

EYE-STRAIN IN HEALTH AND DISEASE. With Special Reference to the Amelioration or Cure of Chronic Nervous Derangements without the Aid of Drugs. By AMBROSE L. RANNEY, A. M., M. D. Illustrated with thirty-eight wood engravings. Philadelphia, New York and Chicago: The F. A. Davis Company, Publishers. 1897.

The well-known author of this work, though his Text-book on Nervous Diseases, Applied Anatomy of the Nervous System, Treatise on Surgical Diagnosis, Practical Medical Anatomy, and the like, needs no introduction to the medical profession, as his works and papers, for some years past, have shown him to be an indefatigable, energetic investigator, seeking to advance the science and art of medicine.

The present volume comprises the substance of several monographs that the author has published from time to time during the past ten years in medical journals, with the addition of considerable new matter. The histories of many typical cases have been added with a view of illustrating some remarkable results of eye treatment alone upon various forms of nervous disturbances that have persisted for years and failed to yield to any other form of treatment. Many of the views which the author advanced in his work on nervous diseases relative to the effects of eye strain upon the development of headache, neuralgia, sleeplessness, chorea, epilepsy, nervous prostration and insanity, are reiterated with strong clinical evidence to sustain them. The author's early convictions are strengthened by the new and large quantity of material presented, and he claims that many of those who were antagonistic to his theories a year ago are now enthusiastic in their support. The author asks his critics to bear in mind three facts—namely, that none of the cases here reported took any drugs while under his care, that they were chronic cases which had received no benefits from medication under skilful hands, and that many of them were made absolutely well by eye treatment alone.

Chapter I. is devoted to the bearings of eye-strain upon the duration of human life, and the keynote of the whole matter is thus enunciated, "that any excess of nervous expenditure to one organ over the normal amount which should be furnished, is done at the expense of the others sooner or later." In this chapter the relation between eye-strain and phthisis is especially considered, the author even citing his own case, because in his maternal ancestry phthisis had been extremely frequent and the duration of life materially lessened thereby. A high degree of latent hypermetropia, whose existence was unsuspected until atropine was instilled into his eyes, marked a turning point in his own physical state, and —the author infers—arrested any further tubercular progress.

The author makes the following surprising statement: "I have never yet encountered a case of typical phthisis in which eye-strain did not exist as a factor more or less potent, in my opinion, in causing and hastening its development." This is a remarkable fact if borne out by other observers and at once decides one of the first duties of the physician towards his tubercular patients. The author also suggests that in the future life insurance examiners will deem it necessary to take into consideration the possible existence of these hidden factors of disease before they pass final judgment.

Chapter II. takes up in a clear and understandable way the various tests of vision and ocular movements, also a translation of the terms so generally employed by ophthalmologists and so easily forgotten by the general practitioner.

Chapter III. is devoted to eye-strain as a cause of headache and neuralgia. Headaches are discussed under three prominent classes: the toxic, organic and reflex varieties. Of the latter the author cites many cases cured by relieving eye-strain.

Chapter IV. is devoted to the eye treatment of chorea. The author asserts that clinical experience has demonstated most positively a direct causal relationship between eye-strain and chorea. Cases are cited where again eye treatment has cured the chorea.

If such relationship does exist how is it possible for the great majority of choreatics to recover without eye treatment and in many cases without any well-defined mode of treatment?

Chapter V. is devoted to sleeplessness; Chapter VI. to eye-strain as a cause of chronic gastric and digestive disturbances; Chapter VII. to the eye treatment of epileptics; Chapter VIII. to the eye treatment of nervous prostration and insanity; Chapter IX. to the surgical treatment of anomalies of the ocular muscles; and in Chapter X. the author gives a few practical hints relative to eye-strain as a cause of abnormal eye conditions.

On the whole, the work is exceedingly instructive and entertaining; the author is an enthusiast on the subject and is happy in his own convictions. It would hardly be a wise policy to turn over indiscriminately to the operating ophthalmologist all cases of general neuroses for treatment, but surely some cases, perhaps many, would be fit candidates for this form of treatment. How a case of paresis, fully developed, of a specific nature is amenable to the graduated tenotomy treatment or prism treatment the reviewer cannot understand.

A good way to test any new rule, law or mode of treatment is to study the failures as well as the successes and then to pass judgment upon the former as well as upon the latter. There are other forms of reflex irritation besides eye-strain and in the treatment of any case of neurosis all organs should be carefully examined and treated.

The subject treated by Dr. Ranney has certainly gained ground and thus is entitled to a conspicuous though not an overshadowing place in the methods or modes of treatment of the functional neuroses.

The publishers have in their usual excellent way added to the value of the book by the careful typographical work bestowed upon it. W. C. K.

